# In-Flight Alignment of Integrated SINS/GPS/Polarization/Geomagnetic Navigation System Based on Federal UKF

**DOI:** 10.3390/s22165985

**Published:** 2022-08-10

**Authors:** Songyin Cao, Honglian Gao, Jie You

**Affiliations:** Department of Automation, College of Information Engineering, Yangzhou University, Yangzhou 225127, China

**Keywords:** inertial navigation system, polarization navigation, integrated navigation, in-flight alignment, unscented Kalman filter

## Abstract

As a common integrated navigation system, the strapdown inertial navigation system (SINS)/global positioning system (GPS) can estimate velocity and position errors well. Many auxiliary attitude measurement systems can be used to improve the accuracy of attitude angle errors. In this paper, the in-flight alignment problem of the integrated SINS/GPS/Polarization/Geomagnetic navigation system is discussed. Firstly, the SINS/Geomagnetic subsystem is constructed to improve the estimation accuracy of horizontal attitude angles. Secondly, the polarization sensor is used to improve the estimation accuracy of heading angle. Then, a federal unscented Kalman filter (FUKF) with non-reset structure is applied to fuse the navigation data. Finally, simulation results for the integrated navigation system are provided based on experimental data. It can be shown that the proposed approach can improve not only the speed and position, but also the attitude error effectively.

## 1. Introduction

The inertial navigation system (INS) is a mature navigation system which can provide complete and continuous navigation parameters, with the advantages of good stealth, not relying on external information, high accuracy in a short period of time, and so on [1,2]. One of the key technologies of INSs is calibration and alignment, which can be classified into stationary base alignment and in-flight alignment [3]. In [4,5], multi-objective robust filtering schemes were proposed to the initial alignment problem of INS with multiple disturbances and sensor faults. As a common integrated navigation system, strapdown INS (SINS)/global positioning system (GPS) can effectively improve velocity and position errors [6,7]. However, the estimation accuracy of attitude angle for the integrated SINS/GPS navigation system should be improved in general [8]. An adaptive estimation algorithm and a strong tracking filter with strong robustness were proposed to adjust the window size of data processing for the integrated INS/GPS system in [9]. An optimization-based coarse alignment approach with aided GPS position/velocity was proposed for the coarse in-flight alignment without any prior attitude information in [10]. A high-accuracy GPS-aided coarse alignment method of SINS was proposed to jointly estimate the attitude matrix between current and initial body frames and the unknown gyro bias, accelerometer bias, and lever arm in [11]. The natural physical field information, such as geomagnetic and polarization, can effectively improve the attitude measurement capability and navigation performance. As an autonomous navigation system, geomagnetic navigation has the advantages of being all-weather, independent, unrestricted by terrain, and so on [12]. It has been widely used in the carriers of space, land, and underwater [13,14,15]. The attitude angles estimation problem can be solved by using the integration of the geomagnetic and inertial measurement unit (IMU). However, the heading angle estimation is not satisfactory due to the low accuracy of geomagnetic sensor [16]. The polarization navigation can provide the precise heading information with the polarization distribution of sky light. As the position of the sun changes, the sky light reflects the stable polarization distribution characteristics in real time. In [17,18], a series of polarization sensors were designed to imitate the polarized light-sensitive structure and the navigation mechanism of insect compound eyes. Polarization navigation has the advantages of no accumulated error, strong autonomy, being less susceptible to external disturbance, and being a simple system [19]. In [20], an autonomous initial alignment method for the stationary SINS with the bio-inspired polarized skylight sensors was proposed to improve the precision and convergence speed. In [21], a polarization compass and GPS were integrated to assist the MEMS-INS in suppressing drift in the heading angle and position measurements. With the development of bionic vision navigation technology, the polarization characteristic-based navigation method has prospects of being widely applied in rapid attitude determination and high-precision autonomous navigation of satellites [22].

One of the key problems for in-flight alignment is information fusion, which can be classified into centralized fusion and decentralized fusion. In general, the centralized fusion approach has high accuracy with large computing load and poor fault tolerance. Compared with the centralized fusion, decentralized fusion is a global suboptimal filtering algorithm with strong fault tolerance [23]. In the complex environment of modern navigation system, the abilities of fault tolerance and reliability become more and more important [24]. As an improved decentralized filtering method, the federal Kalman filter (FKF) has been widely used in linear systems with less algorithmic complexity, enhanced fault tolerance and reliability. In [25], an adaptive FKF method was designed to automatically update the information sharing factor. In [26], an improved federal extended Kalman filter (EKF) was applied to the near-ground short-range navigation of small unmanned aerial vehicle (UAV) to obtain better attitude information. In [27], the federal EKF algorithm was used to fuse navigation data in the UAV monitoring problem. To avoid the truncation error generated by the EKF, in this paper, the UKF method with deterministic sampling is used to solve the nonlinear problem.

The main objective of this paper is to present an in-flight alignment approach for the integrated SINS/GPS/Polarization/Geomagnetic system. Unlike previous works [3,4,5,20], the in-flight alignment problem is considered in this paper. The nonlinear error model of the integrated navigation system is established. GPS can provide the carrier’s geographical location and speed information. When the carrier is in a maneuver, the integrated SINS/GPS navigation system can improve the accuracy of not only the position and velocity errors, but also the attitude angle errors. The higher the accuracy of attitude angle errors, the higher the alignment accuracy. Therefore, the polarization and geomagnetic field information are developed to improve the attitude angle errors and suppress the gyroscopic drift. Compared with the literature [8,9,10,11], a federal UKF with reset-free structure is applied for the in-flight alignment problem in this paper. When the carrier is in a maneuver, the proposed reset-free FUKF with improved fault-tolerant capacity is more reliable than the reset FUKF. In addition, the dimension of the local filter for SINS/GPS is 15, and the dimensions of the other two local filters is only 6 in this paper. The computational burden of the proposed FUKF is reduced.

The rest of this paper is organized as follows. Section 2 introduces the principles of each navigation system and the error model of the integrated navigation system. In Section 3, the process of non-reset federal UKF is explained. Section 4 demonstrates the analysis and summary of experimental results. Conclusions are given in Section 5.

## 2. The Error Model of Integrated Navigation System

### 2.1. Nonlinear Model of SINS

In this paper, the local level East-North-Up (ENU) frame is selected as the navigation frame. The Right-Front-Up (RFU) frame is chosen as the body frame. The origin of the inertial frame is the center of mass of the earth, the *z* axis is the rotation axis of the Earth, pointing to the north pole. The *x* axis points to the vernal equinox in the equatorial plane. The *y* axis forms a right-handed orthogonal frame with the *z* and *x* axis. See [3] for more details.

The attitude errors equation of SINS is formulated as follows, specifically in Reference [28].

(1) $$\dot{\phi }=C_{\omega }^{-1}\left[\left(I_{3*3}-C_{n}^{n^{\prime }}\right)\omega_{in}^{n}+C_{n}^{n^{\prime }}\delta \omega_{in}^{n}-C_{b}^{n^{\prime }}\delta \omega_{ib}^{b}\right]$$

where $\phi ={\left[\begin{array}{ccc} \phi_{x} & \phi_{y} & \phi_{z} \end{array}\right]}^{T}$ are the misalignment angles of the *x* axes, *y* axes and *z* axes, respectively. The gyroscope drift $\delta \omega_{ib}^{b}$ can be denoted as

(2) $$\delta \omega_{ib}^{b}=\varepsilon^{b}+w_{g}^{b}$$

$\varepsilon^{b}={\left[\begin{array}{ccc} \varepsilon_{x} & \varepsilon_{y} & \varepsilon_{z} \end{array}\right]}^{T}$ is gyroscope random bias, $w_{g}^{b}$ is the zero-mean Gaussian white noise of the gyroscope. $\omega_{in}^{n}$ is the projection of the angular velocity of the navigation frame *n* relative to the inertial frame *i* in the navigation frame *n*, $\delta \omega_{in}^{n}$ is the corresponding error. $C_{b}^{n^{\prime }}$ is the posture transformation matrix from the body frame *b* to the computational navigation frame *n*. $C_{\omega }^{-1}$ is the matrix with respect to the three misalignment angles, $C_{n}^{n^{\prime }}$ is the transformation matrix between the navigation frame *n* and computation frame $n^{\prime }$, the specific expressions for $C_{\omega }^{-1}$ and $C_{n}^{n^{\prime }}$ can be written as:

(3) $$C_{\omega }^{-1}=\left[\begin{array}{ccc} \cos\phi_{y} & 0 & \sin\phi_{y}\\ \sin\phi_{y}\tan\phi_{x} & 1 & -\cos\phi_{y}\tan\phi_{x}\\ -\frac{\sin\phi_{y}}{\cos\phi_{x}} & 0 & \frac{\cos\phi_{y}}{\cos\phi_{x}} \end{array}\right]$$

and

(4) $$C_{n}^{n^{\prime }}=\left[\begin{array}{ccc} C_{11} & C_{12} & C_{13}\\ C_{21} & C_{22} & C_{23}\\ C_{31} & C_{32} & C_{33} \end{array}\right]$$

where

(5) $$\left\{\begin{array}{c} C_{11}=\cos\phi_{y}\cos\phi_{z}-\sin\phi_{y}\sin\phi_{x}\sin\phi_{z}\\ C_{12}=\cos\phi_{y}\sin\phi_{z}+\sin\phi_{y}\sin\phi_{x}\cos\phi_{z}\\ C_{13}=-\sin\phi_{y}\cos\phi_{x},\;C_{21}=-\cos\phi_{x}\sin\phi_{z}\\ C_{22}=\cos\phi_{x}\cos\phi_{z},\;C_{23}=\sin\phi_{x}\\ C_{31}=\sin\phi_{y}\cos\phi_{z}+\cos\phi_{y}\sin\phi_{x}\sin\phi_{z}\\ C_{32}=\sin\phi_{y}\sin\phi_{z}-\cos\phi_{y}\sin\phi_{x}\cos\phi_{z}\\ C_{33}=\cos\phi_{y}\cos\phi_{x} \end{array}\right.$$

The velocity errors equation of SINS is defined as:

(6) $$\delta {\dot{V}}^{n}=\left(I_{3\times 3}-C_{n^{\prime }}^{n}\right)C_{b}^{n^{\prime }}f^{b}+C_{b}^{n}\delta f^{b}-\left(2\omega_{ie}^{n}+\omega_{en}^{n}\right)\times \delta V^{n}-\left(2\delta \omega_{ie}^{n}+\delta \omega_{en}^{n}\right)\times V^{n}+\delta g^{n}$$

where $\delta V^{n}={\left[\begin{array}{ccc} \delta V_{E} & \delta V_{N} & \delta V_{U} \end{array}\right]}^{T}$ is velocity error, the notation [ζ×] represents a skew-symmetric matrix of vector ζ. $\delta V_{E}$, $\delta V_{N}$, $\delta V_{U}$ are the velocity errors in eastward, northward and skyward, respectively. The accelerometer bias $\delta f^{b}$ can be represented as:

(7) $$\delta f^{b}=\nabla^{b}+w_{a}^{b}$$

$\nabla^{b}={\left[\begin{array}{ccc} \nabla_{x} & \nabla_{y} & \nabla_{z} \end{array}\right]}^{T}$ is accelerometer zero bias. $w_{a}^{b}$ is the zero-mean Gaussian white noise of the accelerometer. $\omega_{ie}^{n}$ is projection of the earth rate $\omega_{ie}$ in the navigation frame *n*, $\delta \omega_{ie}^{n}$ is the corresponding error. $\omega_{en}^{n}$ is the representation of the angular velocity of the navigation frame *n* with respect to the terrestrial coordinate frame *e*, $\delta \omega_{en}^{n}$ is the corresponding error. $\delta g^{n}$ is the error of gravitational acceleration in the navigation frame *n*.

The position errors equation of SINS is defined as:

(8) $$\delta {\dot{P}}^{n}=A_{P}\delta P^{n}+B_{P}\delta V^{n}$$

where $\delta {\dot{P}}^{n}={\left[\begin{array}{ccc} \delta L & \delta \lambda & \delta h \end{array}\right]}^{T}$ is position error. δL, δλ, δh are the latitude error, longitude error and height error, respectively. Matrices $A_{p}$ and $B_{p}$ can be described by:

(9) $$A_{P}=\left[\begin{array}{ccc} 0 & 0 & \frac{-V_{N}}{{\left(R_{M}+h\right)}^{2}}\\ \frac{V_{E}\tan L\sec L}{R_{N}+h} & 0 & \frac{-V_{E}\sec L}{{\left(R_{N}+h\right)}^{2}}\\ 0 & 0 & 0 \end{array}\right]$$

(10) $$B_{P}=\left[\begin{array}{ccc} 0 & \frac{1}{R_{M}+h} & 0\\ \frac{\sec L}{R_{N}+h} & 0 & 0\\ 0 & 0 & 1 \end{array}\right]$$

where $R_{M}$ denotes the curvature radius of the meridian, $R_{N}$ is the curvature radius of the prime vertical. *L*, λ and *h* are latitude, longitude and height, respectively.

Extending the accelerometer zero bias and gyroscope drift to the error state, based on the Equations (1), (6) and (8), the error equation of SINS can be rewritten as:

(11) $$\left\{\begin{array}{ccc} \dot{\phi } & = & C_{\omega }^{-1}\left[\left(I_{3*3}-C_{n}^{n^{\prime }}\right)\omega_{in}^{n}+C_{n}^{n^{\prime }}\delta \omega_{in}^{n}-C_{b}^{n^{\prime }}\delta \omega_{ib}^{b}\right]\\ \delta {\dot{V}}^{n} & = & \left(I_{3\times 3}-C_{n^{\prime }}^{n}\right)C_{b}^{n^{\prime }}f^{b}+C_{b}^{n}\delta f^{b}-\left(2\omega_{ie}^{n}+\omega_{en}^{n}\right)\times \delta V^{n}-\left(2\delta \omega_{ie}^{n}+\delta \omega_{en}^{n}\right)\times V^{n}+\delta g^{n}\\ \delta {\dot{P}}^{n} & = & A_{P}\delta P^{n}+B_{P}\delta V^{n}\\ {\dot{\varepsilon }}^{b} & = & 0_{3\times 1}\\ {\dot{\nabla }}^{b} & = & 0_{3\times 1} \end{array}\right.$$

The error equation can be further expressed as:

(12) $$\dot{X}\left(t\right)=F\left(X\left(t\right)\right)+W\left(t\right)$$

where system state variable is

$$X={\left[\begin{array}{ccccc} \phi^{T} & \delta V^{T} & \delta P^{T} & \varepsilon^{bT} & \nabla^{bT} \end{array}\right]}^{T}$$

Here F(X) is a nonlinear function. W(t) is the process noise vector.

(13) $$W\left(t\right)={\left[\begin{array}{ccc} {\left(C_{\omega }^{-1}C_{b}^{n^{\prime }}\omega_{g}^{b}\right)}^{T} & {\left(C_{b}^{n}\omega_{a}^{b}\right)}^{T} & 0_{1\times 9} \end{array}\right]}^{T}$$

### 2.2. Measurement Equation of SINS/GPS

The integrated SINS/GPS navigation system adopts a loose combination mode.

(14) $$Z_{1}=\left[\begin{array}{c} {\tilde{P}}_{s}-{\tilde{P}}_{g}\\ {\tilde{V}}_{s}-{\tilde{V}}_{g} \end{array}\right]=\left[\begin{array}{c} \delta P_{s}+\delta P_{g}\\ \delta V_{s}+\delta V_{g} \end{array}\right]$$

The measurement equation of the SINS/GPS can be further derived as:

(15) $$Z_{1}=\left[\begin{array}{ccc} 0_{3\times 6} & I_{3\times 3} & 0_{3\times 6}\\ 0_{3\times 3} & I_{3\times 3} & 0_{3\times 9} \end{array}\right]X+v_{1}$$

where ${\tilde{P}}_{s}$, ${\tilde{P}}_{g}$ are the position measurements of SINS and GPS, respectively. $\delta P_{s}$, $\delta P_{g}$ are the corresponding position measurement errors. ${\tilde{V}}_{s}$, ${\tilde{V}}_{g}$ are the velocity measurements of SINS and GPS, respectively. $\delta V_{s}$, $\delta V_{g}$ are the corresponding velocity measurement errors.

### 2.3. Measurement Equation of SINS/Polarization

During polarized light navigation there often occurs a phenomenon of polarization singularity. Therefore, the sun vector calculated from the polarized light is used as an observation variable in this paper. A set of six mutually perpendicular polarized light sensors is designed to obtain the polarized light vector [20]. The two polarization sensors establish the module frame as $m_{1}$ and $m_{2}$. The transformation matrix between the two modules is known:

(16) $$C_{m_{1}}^{m_{2}}=\left[\begin{array}{ccc} 1 & 0 & 0\\ 0 & 0 & 1\\ 0 & -1 & 0 \end{array}\right]$$

The projections of the solar vector in the frame $m_{1}$ is $S^{m_{1}}$

(17) $$S^{m_{1}}=\pm \frac{1}{\Delta_{1}\cos\left({\tilde{\phi }}_{m_{2}}\right)}\left[\begin{array}{c} \sin\left({\tilde{\phi }}_{m_{1}}\right)\sin\left({\tilde{\phi }}_{m_{2}}\right)\\ -\cos\left({\tilde{\phi }}_{m_{1}}\right)\sin\left({\tilde{\phi }}_{m_{2}}\right)\\ \pm \sin\left({\tilde{\phi }}_{m_{1}}\right)\cos\left({\tilde{\phi }}_{m_{2}}\right) \end{array}\right]$$

where $\Delta_{1}=\sqrt{\sin^{2}\left({\tilde{\phi }}_{m_{1}}\right)+\tan^{2}\left({\tilde{\phi }}_{m_{2}}\right)}$, and ${\tilde{\phi }}_{m_{1}}$, ${\tilde{\phi }}_{m_{2}}$ are the polarization azimuth angles in frames $m_{1}$ and $m_{2}$, respectively. Due to the singularity of the polarization azimuth, four different solar vectors are described in Equation (17). In order to remove the singularity, the solar vector $S_{m_{2}}$ in the frame $m_{2}$ is introduced as follows:

(18) $$S^{m_{2}}=\pm \frac{1}{\Delta_{2}\cos\left({\tilde{\phi }}_{m_{1}}\right)}\left[\begin{array}{c} \sin\left({\tilde{\phi }}_{m_{1}}\right)\sin\left({\tilde{\phi }}_{m_{2}}\right)\\ -\sin\left({\tilde{\phi }}_{m_{1}}\right)\cos\left({\tilde{\phi }}_{m_{2}}\right)\\ \pm \cos\left({\tilde{\phi }}_{m_{1}}\right)\sin\left({\tilde{\phi }}_{m_{2}}\right) \end{array}\right]$$

where $\Delta_{2}=\sqrt{\sin^{2}\left({\tilde{\phi }}_{m_{2}}\right)+\tan^{2}\left({\tilde{\phi }}_{m_{1}}\right)}$. Based on the transformation relation of the solar vector in the two frames, it can exclude the two solar vectors that are unsatisfied conditions. The retained solar vector is computed as

(19) $${\overset{⌢}{S}}^{m_{1}}=\pm \frac{1}{\Delta_{1}\cos\left({\tilde{\phi }}_{m_{2}}\right)}\left[\begin{array}{c} \sin\left({\tilde{\phi }}_{m_{1}}\right)\sin\left({\tilde{\phi }}_{m_{2}}\right)\\ -\cos\left({\tilde{\phi }}_{m_{1}}\right)\sin\left({\tilde{\phi }}_{m_{2}}\right)\\ \sin\left({\tilde{\phi }}_{m_{1}}\right)\cos\left({\tilde{\phi }}_{m_{2}}\right) \end{array}\right]$$

To further remove the directional singularity of the solar vector, the gravity vector is introduced to determine $S^{b}$ as follows:

(20) $$S^{b}=sign(\frac{S^{n}\cdot g^{n}}{C_{m_{1}}^{b}{\overset{⌢}{\;S}}_{m_{1}}\cdot g^{b}})C_{m_{1}}^{b}{\overset{⌢}{S}}_{m_{1}}$$

where

(21) $$C_{m_{1}}^{b}=\left[\begin{array}{ccc} 1 & 0 & 0\\ 0 & \frac{\sqrt{2}}{2} & \frac{\sqrt{2}}{2}\\ 0 & -\frac{\sqrt{2}}{2} & \frac{\sqrt{2}}{2} \end{array}\right]$$

Substituting optional ${\overset{⌢}{\;S}}_{m_{1}}$ into Equation (20) one can obtain a determined $S^{b}.sign(\cdot )$ is the symbolic function. $g^{n},g^{b}$ are the representations of the gravity vector in the frame n and the frame b, respectively. $g^{n}$ can be obtained from the local latitude and longitude. $g^{b}$ can be obtained from the accelerometer output. $S^{n}$ is the solar vector in the navigation frame, which can be obtained according to the astronomical calendar. Ideally, there is $S^{n}=C_{b}^{n}S^{b}$. Owing to the effect of noise, the calculated navigation frame $n^{\prime }$ and the actual geographic navigation frame n do not exactly coincide. In the moving base, the misalignment angles are large. Therefore, the approximation $C_{n}^{n^{\prime }}\approx I\;-\;[\phi \times ]$ does not hold. Considering the measurement error effect of the polarized light sensor, the measurement equation of polarized light can be written as:

(22) $${\overset{∼}{S}}^{b}=C_{n^{\prime }}^{b}C_{n}^{n^{\prime }}S^{n}+v_{2}$$

where $\delta S^{b}={\left[\begin{array}{ccc} \delta S_{x}^{b} & \delta S_{y}^{b} & \delta S_{z}^{b} \end{array}\right]}^{T}$ is the solar vector error in the body frame b. $v_{2}$ is the zero-mean Gaussian white noise corresponding to the measurement error of the polarization sensor. Defining $Z_{2}=C_{b}^{n^{\prime }}{\overset{∼}{S}}^{b}$, $h_{2}(X)=C_{n}^{n^{\prime }}S^{n}$, the measurement equation of SINS/Polarization can be expressed as:

(23) $$Z_{2}=h_{2}(X)+v_{2}$$

### 2.4. Measurement Equation of SINS/Geomagnetic

Assuming that the theoretical geomagnetic vector of a geographic location is $G^{n}$, the magnetic potential of the main magnetic field is defined as ρ [29]:

(24) $$\begin{array}{ccc} \rho (r,\theta ,\lambda ,t) & = & R\sum_{\alpha =1}^{\alpha_{\max}}{\left(\frac{R}{r}\right)}^{\alpha +1}\left\{\sum_{\beta =0}^{\alpha }\left[g_{\alpha }^{\beta }\left(t\right)\cos\left(\beta \right)\lambda \right]\kappa_{\alpha }^{\beta }\left(\cos\theta \right)\right\}\\ \; & \; & +R\sum_{\alpha =1}^{\alpha_{\max}}{\left(\frac{R}{r}\right)}^{\alpha +1}\left\{\sum_{\beta =0}^{\alpha }\left[h_{\alpha }^{\beta }\left(t\right)\sin\left(\beta \right)\lambda \right]\kappa_{\alpha }^{\beta }\left(\cos\theta \right)\right\} \end{array}$$

where *r* is geocentric distance, θ is the deviation angle from the north pole, and λ is longitude. $g_{\alpha }^{\beta }\left(t\right)$ and $h_{\alpha }^{\beta }\left(t\right)$ are Gaussian coefficients. $\kappa_{\alpha }^{\beta }\left(\cos\theta \right)$ is a Legendre function of degree α and order β in Schmidt quasi-normalized form:

(25) $$\begin{array}{ccc} \kappa_{\alpha }^{\beta }\left(\cos\theta \right) & = & \frac{1}{2^{\alpha }\alpha !}{\left[\frac{\varepsilon_{\beta }\left(\alpha -\beta \right)!{\left(1-\cos^{2}\theta \right)}^{\beta }}{(\alpha +\beta )!}\right]}^{1/2}\times \frac{d^{\alpha +\beta }}{d{\left(\cos\theta \right)}^{\alpha +\beta }}{\left(\cos^{2}\theta -1\right)}^{\alpha } \end{array}$$

where $G^{n}$ is the projection of the negative gradient of ρ in the local geographic frame,

(26) $$\begin{array}{cc} G^{n} & ={\left[\begin{array}{ccc} -G_{\theta } & G_{\lambda } & -G_{r} \end{array}\right]}^{T}\\ & ={\left[\begin{array}{ccc} \frac{1}{r}\frac{\partial \rho }{\partial \theta } & -\frac{1}{r\sin\theta }\frac{\partial \rho }{\partial \theta } & \frac{\partial \rho }{\partial r} \end{array}\right]}^{T} \end{array}$$

The geomagnetic vector in the body frame is $G^{b}=C_{n}^{b}G^{n}$. Similar to the above polarized light sensor, considering the presence of the platform error angle and the measurement error of the geomagnetic sensor, the equation is rewritten as:

(27) $$C_{b}^{n^{\prime }}{\tilde{G}}^{b}=C_{n}^{n^{\prime }}G^{n}+v_{3}$$

where ${\tilde{G}}^{b}$ is the measurement value of geomagnetic vector. $v_{3}$ is the zero-mean Gaussian white noise.

Defined $Z_{3}=C_{b}^{n^{\prime }}{\tilde{G}}^{b}$, $h_{3}\left(X\right)=C_{n}^{n^{\prime }}G^{n}$, the measurement equation of the integrated SINS/Geomagnetic system can be written as:

(28) $$Z_{3}=h_{3}\left(X\right)+v_{3}$$

## 3. Federal Unscented Kalman Filter

**Figure 1:**
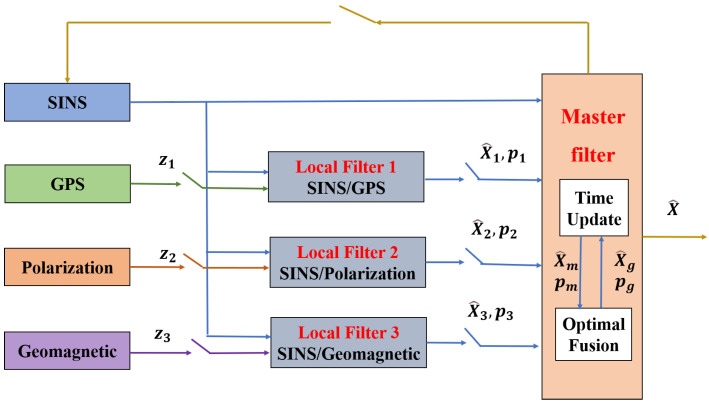
The reset-free federal structure of the integrated SINS/GPS/Polarization/Geomagnetic navigation system.

Due to the unscented transformation, the computational burden of UKF is heavy. In this paper, the measurement equations of both SINS/Polarization and SINS/Geomagnetic are based on the attitude error angles. In order to reduce the computational burden of the integrated navigation system, three attitude error angles and gyroscope drift are used as private states of the SINS/Polarization and SINS/Geomagnetic, except for the full-dimensional state of SINS/GPS. The mode of the federal filtering includes fused-reset, zero-reset, non-reset, and returned structures [27]. In this section, a federal UKF algorithm with non-reset structure is applied for the in-flight alignment problem described in Section 2. See the schematic diagram in Figure 1.

The fourth-order Runge–Kutta method, in the form of numerical integration, is applied to discrete the system model. Then, the discretized equation of error-state for each local system is given as

(29) $$X_{i}\left(k\right)=f_{i}\left(X_{i}\left(k-1\right)\right)+W_{i}\left(k-1\right)$$

where $X_{i}\left(k\right)$, (*i* = 1, 2, 3) is the state vector of each local system. $f_{i}\left(\cdot \right)$ represents the nonlinear function of system state. $W_{i}\left(k\right)\in R^{n_{i}}$ is the process noise which is zero-mean Gaussian white noise with covariance $Q_{i}\left(k\right)>0$.

The discretized measurement equations for each local system are denoted as:

(30) $$Z_{i}\left(k\right)=h_{i}\left(X_{i}\left(k\right)\right)+v_{i}\left(k\right)$$

where $Z_{i}\left(k\right)$, (*i* = 1, 2, 3) are the measurement vectors of each local system. $h_{i}\left(\cdot \right)$ are the nonlinear functions of measurement outputs. $v_{i}\left(k\right)$ is the measured noise which is zero-mean Gaussian white noise with covariance $R_{i}\left(k\right)>0$. The design steps of the federal UKF are as follows.

1. Update Each Local System and Master Filter
  - Set sigma points and weights
    (31) χj,k(i)=X^i(k),j=0χj,k(i)=X^i(k)+ηi[p(k)i]j,j=1,2,⋯,niχj,k(i)=X^i(k)−ηi[p(k)i]j−ni,j=ni+1,⋯,2ni
    (32) $$\begin{array}{c} \left\{\begin{array}{c} \omega_{j}^{\left(i\right)}=\frac{\tau }{\chi_{i}},j=0\\ \omega_{j}^{\left(i\right)}=\frac{1}{2\chi_{i}},j=1,2,\cdots ,2n_{i} \end{array}\right. \end{array}$$
    where $\eta_{i}=n_{i}+\tau$ is used to adjust the sigma points distribution around X^i(k). ω is the mean and covariance weights of the state vector. To prevent the occurrence of negative determinations of the one-step prediction estimation error covariance, the singular value decomposition (SVD) method is adopted to calculate the square root of p(k).
    (33) $$p\left(k\right)=U_{k}S_{k}V_{k}^{T}$$
    and
    (34) $${\left[\sqrt{p(k)}\right]}_{j}=U_{j,k}\sqrt{S_{j,k}}$$
    where $U_{j,k}$ is the jth column of $U_{k}$. $S_{j,k}$ is the jth singular value of $S_{k}$.
  - Time update
    The one-step predicted value of the state vector is described as:
    (35) $$\chi_{j,k+1|k}^{\left(i\right)}=f_{i}\left(\chi_{j,k}^{\left(i\right)}\right),\;\left(i=1,2,\cdots ,m;j=0,1,\cdots ,2n_{i}\right)$$
    (36) X^i(k+1|k)=∑j=02niωj(i)χj,k+1|k(i)
    The one-step prediction mean square error is represented as:
    (37) p^i,xx(k+1|k)=∑j=02niωj(i){[χj,k+1|k(i)−X^i(k+1|k)][χj,k+1|k(i)−X^i(k+1|k)]T}+Qi(k)
    The one-step prediction of the measured value is denoted as:
    (38) $$Z_{i,k+1|k}=h_{i}\left(\chi_{k+1|k}^{\left(i\right)}\right)$$
    (39) $${\hat{Z}}_{i,k+1|k}=\sum_{j=0}^{2n_{i}}\omega_{j}^{\left(i\right)}Z_{j,k+1|k}^{\left(i\right)}$$
  - Measurement update
    Estimated mean squared deviation equation for the measured value is expressed as:
    (40) $$p_{i,zz}\left(k+1|k\right)=\sum_{j=0}^{2n_{i}}\omega_{j}^{\left(i\right)}\left\{\left[Z_{i,k+1|k}-{\hat{Z}}_{i,k+1|k}\right]{\left[Z_{i,k+1|k}-{\hat{Z}}_{i,k+1|k}\right]}^{T}\right\}+R_{i}\left(k\right)$$
    (41) pi,xz(k+1|k)=∑j=02niωj(i){[χj,k+1|k(i)−X^i(k+1|k)][Zi,k+1|k−Z^i,k+1|k]T}
    The state estimation of the master filter is described as:
    (42) X^m,k+1=X^m,k+1|k
    The state estimations covariance of the master filter is denoted as:
    (43) $$p_{m,xx}\left(k+1\right)=p_{m,xx}\left(k+1|k\right)$$
    For three local filters, the filter gains and state updating are computed as
    (44) Ki,k+1=pi,xzpi,zz−1X^i,k+1=X^i,k+1|k+Ki,k+1(Zi,k+1−Z^i,k+1|k)pi,xx(k+1)=pi,xx(k+1|k)−Ki,k+1(Zi,k+1−Z^i,k+1|k)
2. Global Fusion
  The global fusion process only fuses the common states $X_{g}={\left[\begin{array}{cc} \phi^{T} & \varepsilon^{T} \end{array}\right]}^{T}$. Since the local filter of the SINS/GPS is a 15-dimensional state, X^1,k and $p_{1,k}$ must do the corresponding dimensional transformation before data fusion. Among them, only the attitude error angles and gyroscope drift in the estimations of X^1,k are fused. The transformed symbols are written as X^1′ and $p_{1}^{\prime }$, respectively.
  The state of the global fusion is
  (45) $$p_{g}^{-1}=p_{1}^{{}^{\prime }-1}+p_{2}^{-1}+p_{3}^{-1}+p_{m}^{-1}$$
  The covariance of the global fusion is
  (46) X^g=pg(p1′−1X^1′+p2−1X^2+p3−1X^3+pm−1X^m)

In this paper, a federal UKF with non-reset structure is addressed for the in-flight alignment problem of SINS. When the carrier is in a maneuver, the proposed non-reset FUKF with improved fault-tolerant capacity is more reliable than the reset FUKF. In addition, the dimension of the local filter for SINS/GPS is 15, and the dimensions of the other two local filters is only six. The computational burden of the proposed FUKF is reduced.

## 4. Experimental Results and Analysis

**Figure 2:**
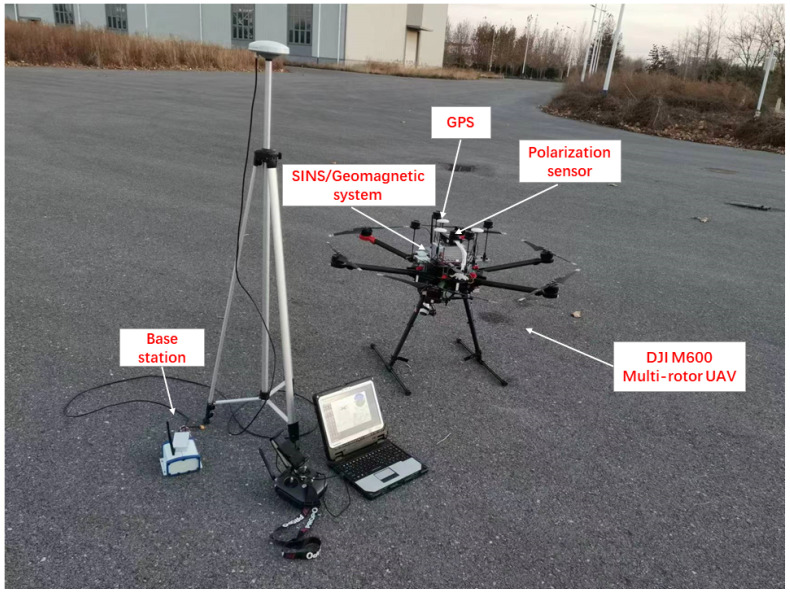
The experimental test of DJI M600 multi-rotor UAV.

In order to evaluate the performance of integrated SINS/GPS/Polarization/Geomagnetic system, the simulation results are based on an experimental test in DJI M600 multi-rotor UAV (see Figure 2). The result is compared with integrated SINS/GPS/Polarization and integrated SINS/GPS/Geomagnetic, respectively. The simulation time is set to 880 s. The parameters of the simulation are shown in Table 1.

The root mean square errors (RMSE) of the attitude, velocity, and position for the three integrated systems are shown in Table 2. Table 3 demonstrates the RMSE of attitude, velocity, and position for the integrated SINS/GPS/Polarization/Geomagnetic system using the FKF and FUKF, respectively.

**Figure 3:**
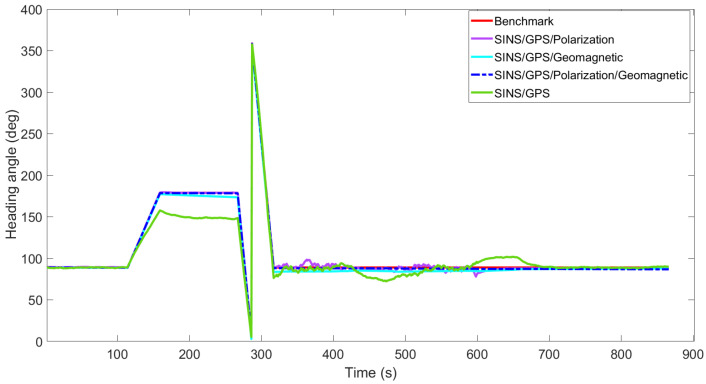
Comparison of yaw angle for different integrated navigation systems.

**Figure 4:**
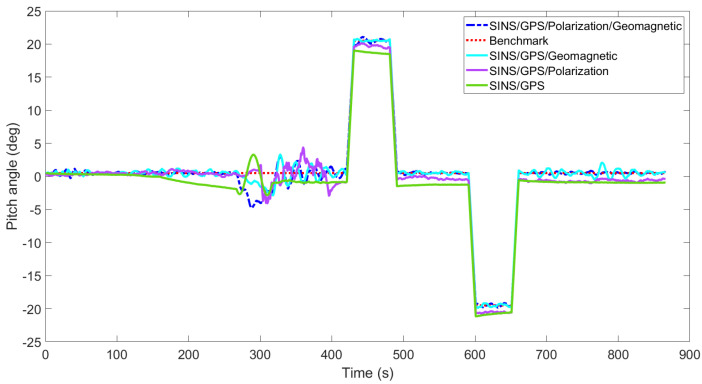
Comparison of pitch angle for different integrated navigation systems.

**Figure 5:**
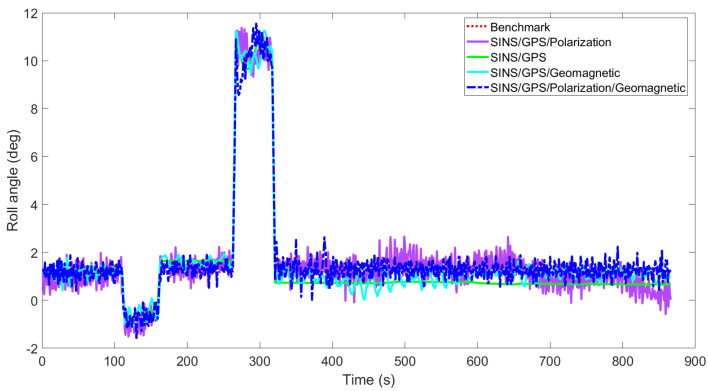
Comparison of roll angle for different integrated navigation systems.

**Figure 6:**
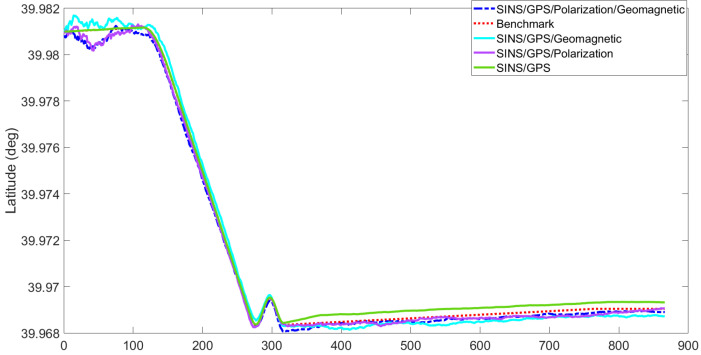
Comparison of latitude for different integrated navigation systems.

**Figure 7:**
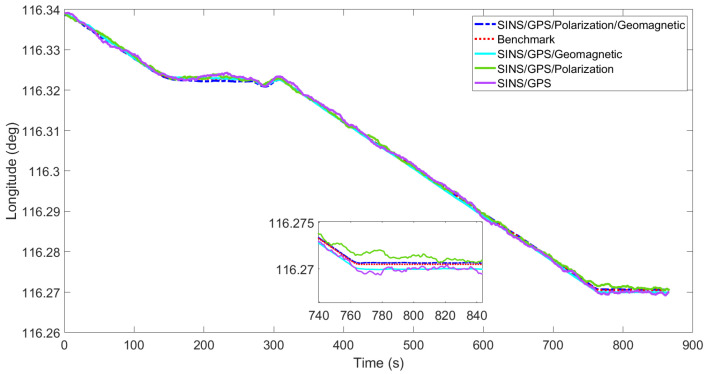
Comparison of longitude for different integrated navigation systems.

**Figure 8:**
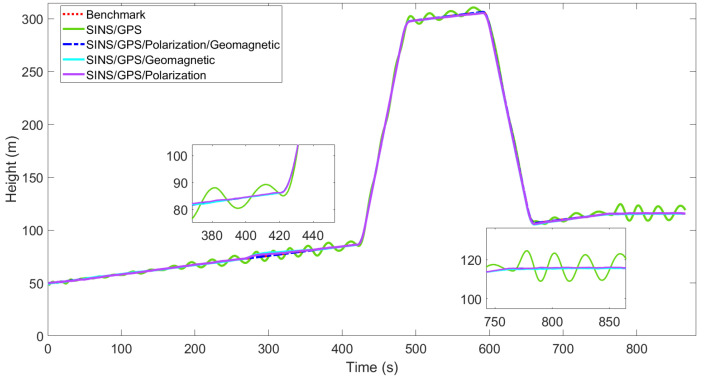
Comparison of height for different integrated navigation systems.

Figure 3, Figure 4, Figure 5, Figure 6, Figure 7 and Figure 8 show the comparison of attitude angles and positions obtained by using FUKF for the four different integrated navigation systems. In Figure 3, Figure 4 and Figure 5, the heading angle of SINS/GPS/Polarization fits the reference value well. However, the pitch and roll angles fluctuate up and down along the reference values. That is because the measurements of accelerometers and gyroscopes contain the carrier’s motion information. The attitude angles measured by accelerometer and gyroscope are not accurate at this time. The addition of polarization information improves heading angle accuracy. The result is consistent with the theory that polarization sensors can measure heading angle well in this paper. Although the accuracy of heading angle of the SINS/GPS/Polarization/Geomagnetism is worse than that of SINS/GPS/Polarization, both pitch and roll angles are better than the other two integrated navigation systems. In Table 2, the SINS/GPS/Polarization has high accuracy of heading angle, while SINS/GPS/Geomagnetic has high accuracy of pitch and roll angles. The geomagnetic sensor is sensitive; the heading angle sometimes deviates from the reference value due to the disturbed magnetic field. Then, the heading angle will be estimated by the gyroscopes. The integration of polarization and geomagnetic makes a compromise for heading angle. Although the accuracy of heading angle of the integrated SINS/GPS/Polarization/Geomagnetic system is slightly worse than that of the SINS/GPS/Polarization, the accuracy of the remaining navigation parameters are higher than that of the other two integrated navigation systems. In Figure 6, Figure 7 and Figure 8, the comparison of position trajectory for the three different integrated navigation systems are demonstrated. From Figure 6, Figure 7 and Figure 8, the position trajectory of the proposed method is better than the counterparts of the SINS/GPS/Polarization navigation system and SINS/GPS/Geomagnetic navigation system. The reason is the polarization and geomagnetism improve the estimation accuracy of attitude angles. Therefore, the posture transformation matrix $C_{b}^{n}$ calculated from the attitude angles is more accurate. During the position update, the position accuracy is improved accordingly. The conclusions of Figure 3, Figure 4, Figure 5, Figure 6, Figure 7 and Figure 8 are consistent with data comparison in Table 2. The experiment results show the effectiveness of SINS/GPS/Polarization/Geomagnetic in the flight of an UAV.

**Figure 9:**
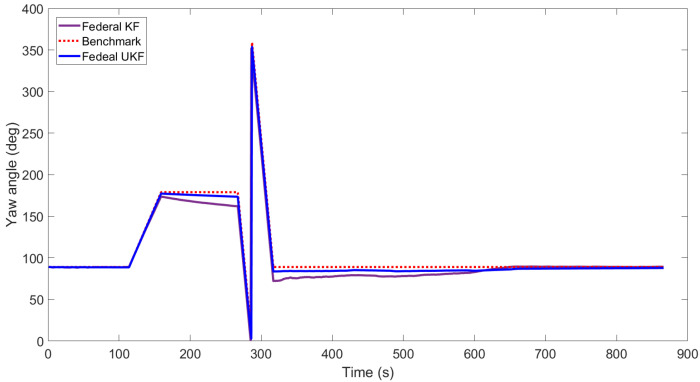
Comparison of yaw angle for integrated SINS/GPS/Polarization/Geomagnetic by FUKF and FKF.

**Figure 10:**
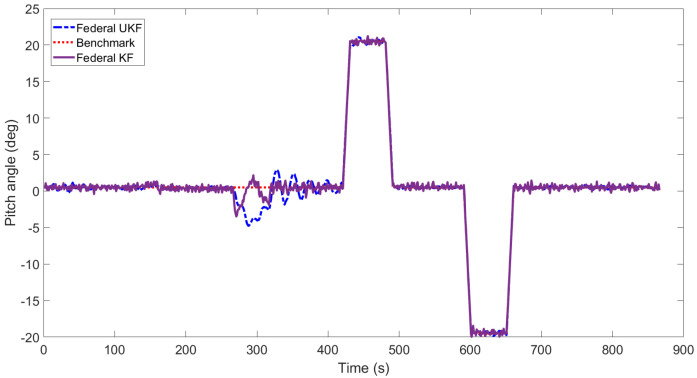
Comparison of pitch angle for integrated SINS/GPS/Polarization/Geomagnetic by FUKF and FKF.

**Figure 11:**
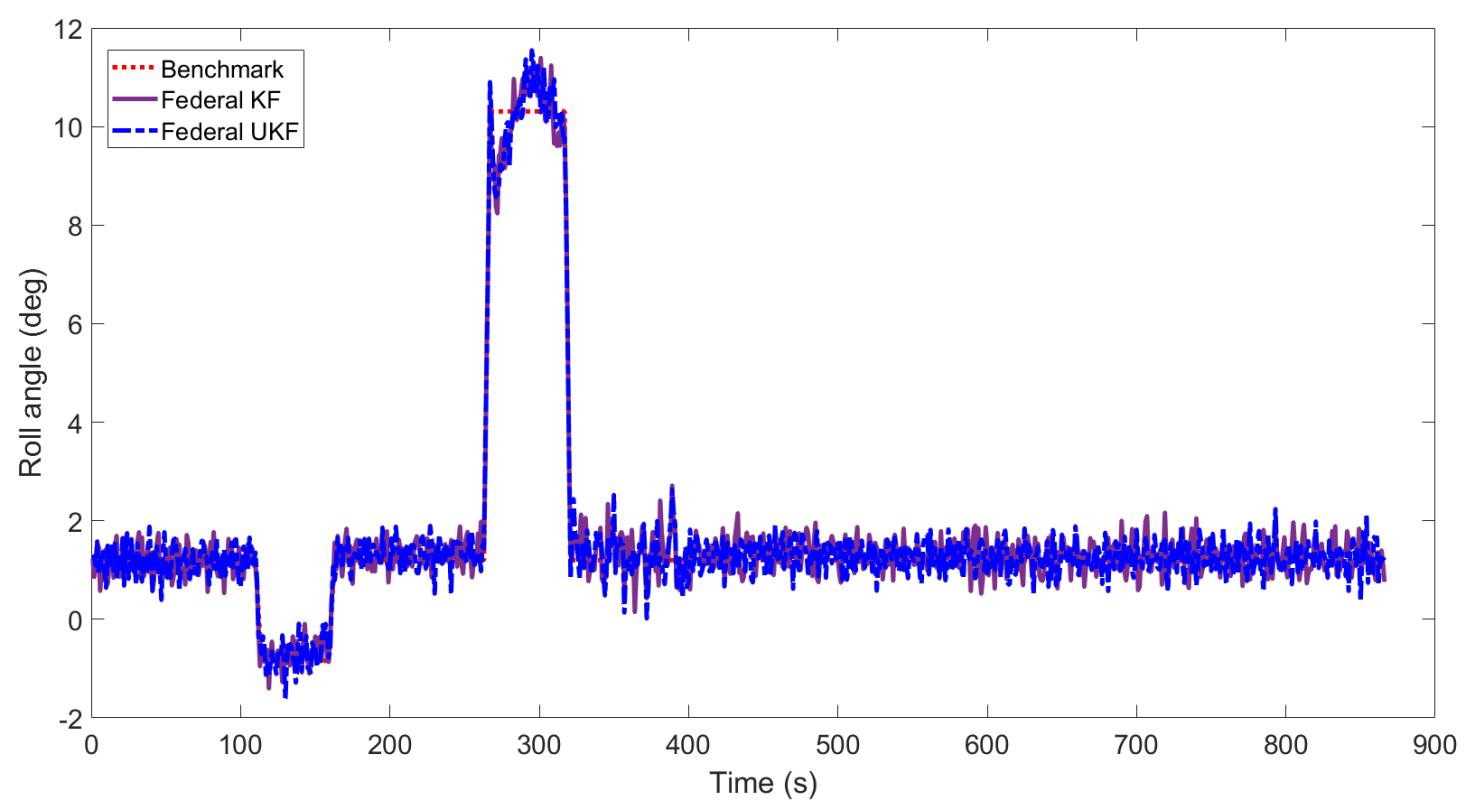
Comparison of roll angle for integrated SINS/GPS/Polarization/Geomagnetic by FUKF and FKF.

Figure 9, Figure 10 and Figure 11 show the attitude angles comparison of the integrated SINS/GPS/ Polarization/Geomagnetic navigation system based on the FKF and FUKF, respectively. It can be seen that the accuracy of attitude angles obtained by the FUKF are better than those of the FKF. In Figure 9, the heading angle obtained by the FUKF is better than the counterpart of FKF. In Figure 10 and Figure 11, the difference between the pitch and roll angles obtained by FUKF and FKF is not significant. Table 3 shows the RMSE of attitude, velocity, and position of the integrated SINS/GPS/Polarization/Geomagnetic navigation system using the FKF and FUKF, respectively. It can be seen that the accuracy of the navigation parameters using the FUKF are higher than those using the FKF, especially in the heading angle.

## 5. Conclusions

This paper discusses the in-flight alignment problem of the integrated SINS/GPS/ Polarization/Geomagnetic navigation system. In the integrated SINS/GPS navigation system, GPS provides velocity and position measurements, which leads to a poor estimation of attitude angle errors. Different from the previous results, this paper proposes an approach to improve the accuracy of attitude angle errors by using auxiliary attitude measurement system.

- Firstly, the SINS/Geomagnetic subsystem is constructed to improve the estimation accuracy of horizontal attitude angles.
- Secondly, aiming at the problem that the heading angle calculated by geomagnetic information is inaccurate in a moving base, the polarization sensor is used to improve the estimation accuracy of heading angle.
- Thirdly, a federal unscented Kalman filter with reset-free structure is proposed for the in-flight alignment problem of the integrated SINS/GPS/Polarization/Geomagnetic navigation system. In the local filter, the unscented Kalman filter is used to estimate the state of each subsystem. In the master filter, attitude angles and gyro drift of geomagnetic and polarization subsystems are estimated to improve the filtering accuracy with low computational burden.

## Figures and Tables

**Table 1:** The parameters of the integrated navigation system.

Initial Attitude	Pitch	0.4985 deg
	Roll	1.1975 deg
	Yaw	88.9975 deg
Initial Velocity	$V_{E}$	0 m/s
	$V_{N}$	0 m/s
	$V_{U}$	0 m/s
Initial Position	Longitude	116.2705 deg
	Latitude	39.9690 deg
	Altitude	115.5942 m
Attitude Error	Pitch	1 deg
	Roll	1 deg
	Yaw	2 deg
Velocity Error	$V_{E}$	1 m/s
	$V_{N}$	1 m/s
	$V_{U}$	1 m/s
Position Error	Longitude	10 m
	Latitude	10 m
	Altitude	10 m
Gyro Parameters	Constant Drift	5.1 deg/h
	Random Walk Coefficient	0.26 deg/$\sqrt{h}$
Accelerometer Parameters	Constant Drift	0.07 mg
	Random Walk Coefficient	0.029 m/s/$\sqrt{h}$
GPS Parameters	Velocity Error	0.05 m/s
	Horizontal Position Error	2.5 m
	Altitude Error	0.025 m
Polarization Parameters	Solar Azimuth	102.0630 deg
	Solar Zenith	45.5189 deg
	Sensor Accuracy	0.1 deg
	Sampling Rates	100 Hz
Geomagnetic Parameters	Sensor Accuracy	0.1 nT
	Sampling Rates	100 Hz

**Table 2:** RMSE of attitude, velocity and position by SINS/GPS/Geomagnetic/Polarization, SINS/GPS/Geomagnetic and SINS/GPS/Polarization.

		SINS/GPS /Geomagnetic /Polarization	SINS/GPS /Geomagnetic	SINS/GPS /Polarization
Attitude (deg)	Pitch	0.5209	1.2046	3.2200
	Roll	0.3692	1.1026	2.7319
	Yaw	6.52670	24.6654	1.9905
Velocity (m/s)	$V_{E}$	0.0800	0.0850	0.0965
	$V_{N}$	0.0808	0.0872	0.0936
	$V_{U}$	0.0228	0.0610	0.2523
Position	Longitude (deg)	0.0002	0.0004	0.0040
	Latitude (deg)	0.0003	0.0003	0.0056
	Altitude (m)	0.4248	0.8345	2.8849

**Table 3:** RMSE of attitude, velocity and position for SINS/GPS/Geomagnetic/Polarization by FUKF and FKF.

		Federal UKF	Federal KF
Attitude (deg)	Pitch	0.5443	0.9658
	Roll	0.3691	0.4293
	Yaw	6.5267	10.1535
Velocity (m/s)	$V_{E}$	0.0800	0.3572
	$V_{N}$	0.0808	0.3232
	$V_{U}$	0.0228	0.1376
Position	Longitude (deg)	0.0002	0.0002
	Latitude (deg)	0.0003	0.0002
	Altitude (m)	0.4248	8.9336

## Data Availability

Not applicable.
